# Role of Rho in Salt-Sensitive Hypertension

**DOI:** 10.3390/ijms22062958

**Published:** 2021-03-15

**Authors:** Wakako Kawarazaki, Toshiro Fujita

**Affiliations:** Research Center for Advanced Science and Technology, Division of Clinical Epigenetics, The University of Tokyo, Meguro-ku, Tokyo 153-8904, Japan; Toshiro.FUJITA@rcast.u-tokyo.ac.jp

**Keywords:** Rho, Rac, salt-sensitive hypertension, salt, blood pressure, angiotensin II, vascular, Wnt, aging, nitric oxide

## Abstract

A high amount of salt in the diet increases blood pressure (BP) and leads to salt-sensitive hypertension in individuals with impaired renal sodium excretion. Small guanosine triphosphatase (GTP)ase Rho and Rac, activated by salt intake, play important roles in the pathogenesis of salt-sensitive hypertension as key switches of intracellular signaling. Focusing on Rho, high salt intake in the central nervous system increases sodium concentrations of cerebrospinal fluid in salt-sensitive subjects via Rho/Rho kinase and renin-angiotensin system activation and causes increased brain salt sensitivity and sympathetic nerve outflow in BP control centers. In vascular smooth muscle cells, Rho-guanine nucleotide exchange factors and Rho determine sensitivity to vasoconstrictors such as angiotensin II (Ang II), and facilitate vasoconstriction via G-protein and Wnt pathways, leading to increased vascular resistance, including in the renal arteries, in salt-sensitive subjects with high salt intake. In the vascular endothelium, Rho/Rho kinase inhibits nitric oxide (NO) production and function, and high salt amounts further augment Rho activity via asymmetric dimethylarginine, an endogenous inhibitor of NO synthetase, causing aberrant relaxation and increased vascular tone. Rho-associated mechanisms are deeply involved in the development of salt-sensitive hypertension, and their further elucidation can help in developing effective protection and new therapies.

## 1. Introduction

Hypertension is the most important risk factor for stroke, heart disease, and kidney disease, with serious implications for life expectancy and disability [1]. Globally, 1.13 billion people, 22% of adults aged over 18 years old, have hypertension, which caused an estimated 9.4 million deaths in 2010 [2]. Many epidemiological studies and surveys showed that high salt intake can cause the development of hypertension [3,4]. There are two types of hypertensive patients: those whose blood pressure (BP) increases significantly with high salt intake, called salt-sensitive, and those whose BP does not, called salt-resistant [5,6,7]. Salt-sensitive individuals have impaired sodium excretion from the kidneys, which leads to salt retention in the body, increased circulating blood volume, increased cardiac output, and ultimately increased peripheral vascular resistance, resulting in increased BP [6,7,8,9,10,11]. To compensate for the increased intravascular volume by salt intake and maintain BP, renal and peripheral vascular resistance decreases, renal blood flow (RBF) increases, and renal sodium excretion increases in salt resistance. However, the decline in peripheral vascular resistance with a high-salt diet is absent in salt-sensitive objects, which is associated with impaired vascular endothelial function and abnormally enhanced vasoconstrictor reaction in vascular smooth muscle cells (VSMCs) [12,13,14]. Factors that affect renal sodium absorption, and thus salt-sensitive hypertension, include the renin-angiotensin-aldosterone system (RAAS) [15], angiotensin II (Ang II) [16,17], aldosterone [18], and the sympathetic nervous system (SNS) [19,20,21]. Ang II causes sodium retention by both increasing tubular sodium reabsorption [16] and by decreasing RBF [22,23]. Aldosterone excess, as observed in aldosterone-treated animal models [24] and obesity [25], promotes sodium reabsorption from the epithelial sodium channel (ENaC) in the distal tubule via the activation of mineralocorticoid receptors (MRs) during salt intake, resulting in fluid retention and salt-sensitive hypertension.

In obesity, there is a promotion of aldosterone-releasing factor production in adipocytes [26,27,28,29], resulting in hyperaldosteronism that is not completely suppressed by salt intake [25], abnormally enhanced RAAS, and SNS activation [20,30], which enhances renal sodium reabsorption and leads to salt-sensitive hypertension. If the feedback action of the normal RAAS is functioning, high salt intake should suppress the RAAS and lower plasma aldosterone levels, thus lowering BP. However, MR activation occurred despite low plasma aldosterone levels in some rodent models of salt-sensitive hypertension, and there was a novel mechanism in which the small guanosine triphosphatase (GTP)ase Rac1 activated MRs, leading to salt-sensitive hypertension and renal impairment [15,31,32]. Thus, Rac1 plays an important role in the development of salt-sensitive hypertension [15,31,32,33,34,35,36]. On the other hand, salt-sensitive hypertension is also caused by changes in homeostasis that vary with life stage [37], such as maternal malnutrition in fetal life [38], obesity in adult life [39], and aging [13]. Although hypertension increases with age and salt sensitivity is enhanced [40,41], the mechanism of age-related hypertension has not been fully elucidated. Recently, we reported that the pathogenesis of age-related hypertension involves the activation of vascular Wnt-RhoA signaling induced by high salt intake [13]. Ang II-induced hypertension requires Rho activation [42,43], and enhanced sensitivity to Ang II via the Wnt-RhoA pathway in aged vasculature contributes to aging-associated salt-sensitive hypertension [13]. In this way, a number of molecular mechanisms triggered by Rho activation are involved in salt-sensitive hypertension [44,45,46,47,48]. RhoA and its downstream effector, Rho-associated protein kinase (ROCK), constitute the RhoA/ROCK system and play important roles in cellular physiological processes, including contraction, focal adhesions, migration, and proliferation. The activation of the RhoA/ROCK pathway plays a pivotal role in the pathogenesis of hypertension and cardiovascular-renal diseases through interaction with Ang II, oxidative stress, and nitric oxide (NO) [49,50].

Although both Rac and Rho, belonging to the same Rho family, are small proteins, they deeply implicate the mechanism of salt-sensitive hypertension. In this review, we focus on the role of Rho in the pathogenesis of salt-sensitive hypertension, with occasional contrast to that of Rac.

## 2. Molecular Mechanisms Regulating Rho and Rac Activation

The Rho family small GTP binding protein, what is called Rho GTPase, is part of the Ras superfamily and has important functions in regulating intracellular signaling and cell morphology. Once the Rho family is activated by various extracellular stimuli, they ultimately control gene transcription and influence fundamental processes such as cell proliferation and differentiation [51].

The Rho family is involved in signaling networks regulating the actin-based cytoskeleton, which drives cell migration, phagocytosis, endocytosis, morphogenesis, and cytokinesis [52]. The Rho family is also involved in cell cycle progression and gene expression, cell polarity [53], hematopoiesis [54], and Wnt signaling [13,55].

**Figure 1:**
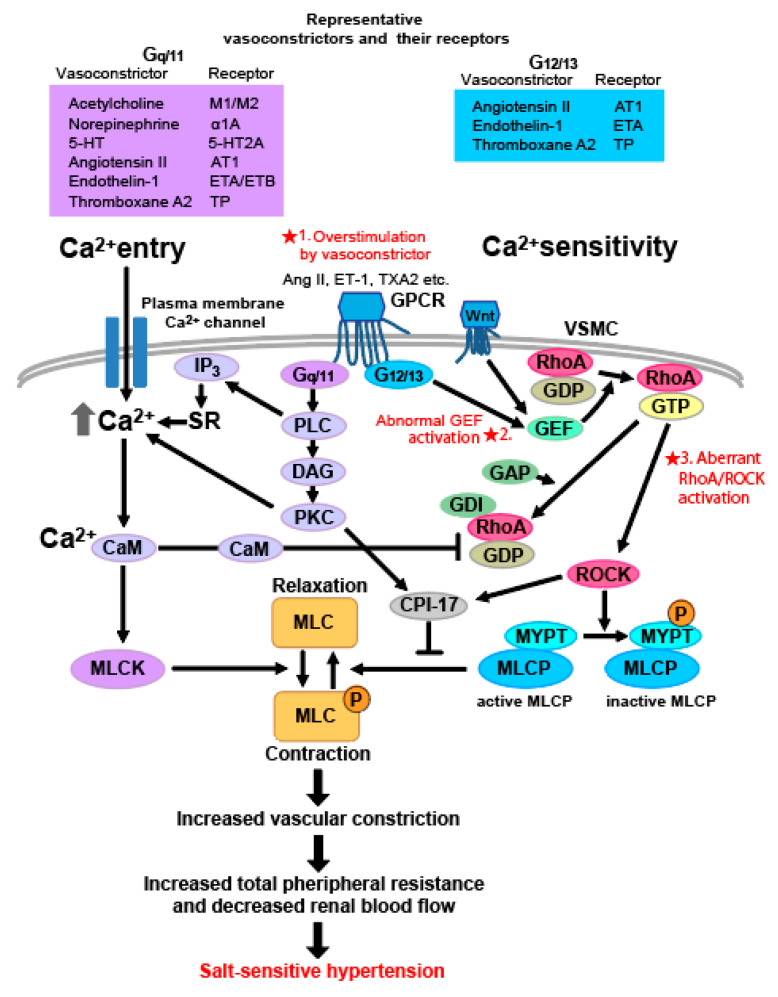
Mechanism of Rho-associated salt-sensitive hypertension in vascular smooth muscle cells (VSMCs) and representative vasoactive peptide coupling to G-protein-coupled receptors (GPCRs) G_q/11_ and G_12/13_. There are three mechanisms of Rho-associated salt-sensitive hypertension in VSMCs. These include the stimulation of VSMCs by increased responsible vasoconstrictors such as angiotensin II (Ang II), endothelin-1 (ET-1), and thromboxane A2 (TXA2) in the blood (★1), abnormal activations of Rho-guanine nucleotide exchange factors (GEFs) (★2), and aberrant activation of Rho/Rho-associated protein kinase (ROCK) by, for example, Wnt noncanonical pathway in aging (★3). These, via Ca^2+^ influx into the cells (★1) and increased Ca^2+^ sensitivity (★1–3), induce VSMCs contraction and increase vasoconstriction, which increases peripheral vascular resistance and decreases renal blood flow, resulting in salt-sensitive hypertension. In VSMC, phosphorylation of myosin light chains (MLCs) is induced by two mechanisms, intracellular Ca^2+^ entry and Ca^2+^ sensitivity. While MLC is phosphorylated by Ca^2+^/calmodulin-dependent MLC kinase (MLCK), it is dephosphorylated by Ca^2+^-independent MLC phosphatase (MLCP) [60]. The receptors binding vasoactive peptides including norepinephrine, acetylcholine, serotonin, Ang II, ET-1, and TXA2 are coupled to GPCRs G_q_ and G_11_, and the subsequent stimulation of phospholipase C (PLC)-β and generation of diacylglycerol (DAG) and protein kinase C (PKC) activates numerous plasma membrane Ca^2+^ channels and promotes calcium influx resulting in the Ca^2+^/calmodulin-dependent activation of MLCK [61]. Stimulated PLC-β also stimulates the second messenger inositol triphosphatase (IP_3_) and induces the intracellular Ca^2+^ release from the sarcoplasmic reticulum (SR). The receptors of vasoconstrictors such as Ang II, ET-1, and TXA2 are coupled to G_12_ and G_13_ to activate the Rho-associated protein kinase (ROCK) pathway, resulting in the inhibition of myosin phosphatase [62,63]. G_12_- and G_13_-mediated activation of RhoA is induced by a subgroup of Rho GEFs, which are activated directly through the interaction with G_α12_ and G_α13_ [64]. GEFs promote the exchange of guanosine diphosphate (GDP) to guanosine triphosphatase (GTP). The inactivation of the GTPase is induced by the GTPase-activating proteins (GAPs). Rho GDP-dissociation inhibitor (GDI) inhibits dissociation of GDP from Rho and hydrolysis of GTP by GAP and keeps Rho in an inactive state [58]. In the Ca^2+^ sensitization of myosin phosphorylation, Rho activates ROCK, and ROCK stimulates phosphorylation of myosin phosphatase target subunit 1 (MYPT1), a regulatory subunit of MLCP. Phosphorylation of MYPT1 inhibits the binding of MLCP to MLC and sustains MLCP phosphorylation to promote contraction. ROCK also inhibits MLCP by phosphorylating the C-kinase-enhancing protein phosphatase 1 inhibitor molecular weight 17 kDa (CPI-17), a smooth muscle-specific inhibitor of MLCP that is activated via the DAG-PLC-PKC pathway [65].

Among Rho GTPases, Rho (RhoA,C), Rac (Rac1,2), and cell division control protein 42 homolog, also known as Cdc42, are by far the best characterized. Focusing on signal transduction, Rho and Rac act as important molecular switches to regulate signal-transduction pathways by interconverting between inactive guanosine diphosphate (GDP)-bound and active GTP-bound conformational states [56]. Switching from GDP-bound to GTP-bound states is tightly controlled by a family of regulatory proteins specific to each Rho GTPase [57]. Guanine nucleotide exchange factors (GEFs) interact with Rho GTPase and promote the exchange of GDP to GTP (Figure 1). Rho GTPase inactivation is induced by the binding of GTPase-activating proteins (GAPs) through increasing GTPase intrinsic hydrolysis activity. On the other hand, the Rho GDP-dissociation inhibitor (GDI) inhibits not only GDP dissociation from Rho GTPase but intrinsic and GAP-stimulated hydrolysis of GTP, keeping Rho GTPase in an inactive state through extracting Rho GTPases from membranes [58]. The substrate specificity of Rho GDI is lower than that of Rho GEF and Rho GAP, and it acts on a wide range of Rho family members [59].

Rho GDIα is highly expressed in the cortical collecting ducts of mice and rats. For example, in Rho GDIα knockout mice, when fed a normal diet, BP was normal, but renal damage was pronounced, and even though serum aldosterone, a ligand for MR, was normal, MR signaling was activated in the kidney, and at the same time Rac1, but not RhoA, was activated [31]. The in vitro experiments provided new evidence that Rac1 activates MR in an aldosterone-independent manner [31]. Thus, the renal injury in Rho GDI knockout mice was caused by the activation of MR by activated Rac1 in the kidney, and MR antagonist eplerenone and Rac1 inhibitor significantly suppressed renal injury [31]. Moreover, when Rho GDIα knockout mice were fed a high-salt diet, they exhibited salt-sensitive hypertension and worsened renal injury, which was mediated by the activated Rac1 and sequential activation of MR signaling in the kidney and was also suppressed by MR antagonist and Rac1 inhibitor [32]. The Rac1-specific GEF, Tiam1, was activated in the kidney of high-salt-fed Rho GDIα knockout mice. Rho-GDIα is known to inhibit both the basal and GEF-stimulated dissociation of GDP from the GDP-bound Rho GTPase and keeps it in the inactive form [66]. Putting all things together, it was suggested that Tiam1, which is activated by dietary high salt intake, modulates salt sensitivity of BP via the activation of Rac1 in salt-fed Rho GDIα mice [32]. Supporting these results, high-salt diet-fed Dahl salt-sensitive (DS) rats showed salt-sensitive hypertension and renal injury via renal MR activation though plasma aldosterone concentrations were suppressed, and they were accompanied by Rac1-GEF and Rac1 activation in the kidney [32]. MR antagonist and Rac1 inhibitor suppressed salt-sensitive hypertension and renal injury by suppressing MR signaling in salt-fed DS rats. However, hypertension and renal injury induced by Rac1-GEF, Rac1, and sequential MR activation could not be observed in high-salt-fed Dahl salt-resistant (DR) rats, salt-fed adrenalectomized DS rats, and low-salt-fed DS rats. These observations suggested that, in vivo, Rac1 activation via Rac1-GEF needs both aldosterone and high salt intake to modulate BP salt sensitivity and cause salt-sensitive hypertension and renal injury [32]. In this way, Rho GEF receives the various stimulation from the outside and regulates the active status of Rho GTPase to convert to intracellular signaling.

## 3. Renin-Angiotensin-Aldosterone System (RAAS) and Salt-Sensitive Hypertension

Essential hypertension is caused by hereditary and environmental factors [37,67]. Many studies have shown that RAAS is closely related to the development of hypertension along with salt intake [68]. In the regulation of the RAAS, with low salt intake, renin is secreted from the juxtaglomerular apparatus of the kidney, sensing a decrease in renal perfusion pressure, sympathetic nerve excitation, and a decrease in sodium concentration in blood. Renin, a protease, acts on angiotensinogen in the blood to release angiotensin I, which is converted to Ang II by angiotensin-converting enzyme (ACE), which is abundant in the endothelial cell membranes of the lungs or kidneys. Ang II is a potent vasoconstrictor, increases aldosterone production by the adrenal glands, and promotes sodium reabsorption in the renal tubules, thereby increasing BP. When circulating plasma volume increases and BP rises, negative feedback suppresses renin secretion, thereby inhibiting RAAS function. Thus, the RAAS maintains circulating plasma volume and regulates BP, and high salt intake suppresses the RAAS.

There are individual differences in the susceptibility of BP elevation by high salt intake, and possibly related to inherited susceptibility [69]. Patients with essential hypertension are divided into two groups: salt-sensitive and salt-resistant. It is reported that the former increases BP by 10% or more when changing from a low-salt diet (Na 0.5 g/day) to a high-salt diet (Na 14.7 g/day) [6].

According to Guyton et al., the pathogenesis of salt-sensitive hypertension is closely related to impaired renal sodium excretion [70]. They demonstrated that the pathogenic mechanism of salt-sensitive hypertension was that high salt intake initially resulted in fluid retention and increased BP due to increased cardiac output because of impaired renal sodium excretion, but then cardiac output decreased while BP increased, and finally vascular autoregulatory changes were observed that increased vascular resistance, maintaining BP elevation in the long term [8,70]. In fact, high salt intake lowers renal vascular resistance and increases RBF in patients with salt-resistant hypertension, while it increases renal vascular resistance and decreases RBF in patients with salt-sensitive hypertension [9]. In other words, RAAS is stimulated and not fully suppressed in salt-sensitive hypertensive patients due to the decrease in renal perfusion during high salt intake.

In DS rats, unlike DR rats, plasma angiotensinogen was decreased, but intrarenal angiotensinogen was increased during high salt intake [71]. Thus, there is a paradoxical enhancement of RAAS in the kidney in the development of salt-sensitive hypertension. The sequential increase in Ang II not only stimulates the production of aldosterone but also has a strong vasoconstrictive effect and promotes sodium reabsorption in the renal tubules [16,17]. Aldosterone also stimulates sodium reabsorption in the renal tubules [18]. However, in obesity, aldosterone production is not sufficiently suppressed even under high salt intake because aldosterone production is stimulated by the secretion of aldosterone-releasing factor from the adipocytes [25]. Furthermore, the activation of RhoA is necessary for the vasoconstrictor effect of Ang II [42], and the activation of Rho GEF and RhoA in blood vessels is involved in the increased sensitivity to Ang II in salt-sensitive hypertension [13,72]. Thus, in salt-sensitive hypertension, there is inappropriate activation of RAAS that activates RhoA/ROCK, which is closely related to the development of salt-sensitive hypertension.

## 4. Central Nervous System

### Role of Rho and Rac1 in Salt-Sensitive Hypertension

The autonomic nervous system, centered on the arterial baroreceptor reflex, plays a pivotal role in maintaining BP [73,74]. Instantaneous BP is sensed by baroreceptors, which are stretch receptors located in the carotid sinus and aortic arch, and this information is transmitted via afferent nerve fibers to the nucleus of the solitary tract (NTS) in the brainstem; then, it alters the excitability of the rostral ventrolateral medulla (RVLM) via intervening nerves, regulating BP [75]. The RVLM projects postganglionic sympathetic fibers to various organs via the spinal cord and the sympathetic ganglia. Moreover, the RVLM receives information from higher centers of BP regulation, such as the subfornical organ (SFO), the supraoptic nucleus (SON), and the paraventricular nucleus (PVN) of the hypothalamus, and integrates sympathetic output [75]. In vivo studies in rats have shown that RhoA/ROCK in the NTS plays a critical role in SNS-mediated maintenance of basal BP [76]. In addition, transfection in rat NTS with an adenoviral vector encoding a dominant-negative Rho kinase or local microinjection of the Rho-kinase inhibitor Y27632 reduces mean BP and urinary norepinephrine excretion [76]. These contrast with the fact that inhibition of the Rac1 signaling pathway in the NTS has no effect on basal BP [77].

On the other hand, high salt intake affects RhoA and Rac1 in the central nervous system, leading to salt-sensitive hypertension. Salt intake induces sympathoexcitation via the central nervous system [30,78,79], which involves ENaCs in the brain [80]. High salt intake also increases the concentration of Na^+^ in the cerebrospinal fluid (CSF), leading to the activation of brain renin-angiotensin system (RAS) and increased Ang II production, which increases sympathetic nerve activity (SNA) and induces vasoconstriction [81,82,83]. It has been shown that a high-salt diet elevated the Na^+^ of the CSF in DS rats but not in salt-resistant strains such as Wistar, Wistar-Kyoto (WKY), and Dahl salt-resistant rats [84,85]. As the primary effector peptide of the RAAS, Ang II, binding to the Ang II Type 1 receptor (AT1R), contributes to BP elevation via brain nuclei such as PVN and SON in salt-fed DS rats [86]. In mice with pressure overload produced by aortic banding, high salt intake induced increased sympathetic outflow, cardiac dysfunction, and salt-sensitive hypertension, but not in sham-operated mice [46]. The intracerebroventricular (ICV) infusion of high-Na artificial CSF also induced similar changes to those of high salt intake. However, the ICV infusion of ENaC blocker benzamil to high-salt-fed mice with pressure overload suppressed the augmented sympathetic outflow and improved cardiac function. They were also suppressed by injection of AT1R blockers or Rho-kinase inhibitors into the ICV. These results suggested that mice with pressure overload acquired brain Na sensitivity due to the activation of ENaCs in the brain by stimulating the Rho/ROCK and Ang II-AT1R pathway, which leads to salt-induced sympathetic hyperactivation and further increased BP, resulting in pressure overload and cardiac dysfunction [46].

Furthermore, brain RAS could regulate oxidative stress in the PVN in the development of hypertension. Rac1 constitutes and activates NADPH oxidase, which is essential for superoxide production [87]. And, it has also been shown that increased Ang II in the brain induces NADPH-dependent reactive-oxygen-species (ROS) production by protein kinase C (PKC), which is an important process in the activation of Rac1 and subsequent enzyme assembly [88,89]. In Wistar rats, high salt intake induced hypertension, increased levels of AT1R, PKCγ, and Rac1 activity and superoxides, but decreased the levels of antioxidants in the PVN compared to those in the control animals [34]. The PVN infusion of AT1R antagonist losartan to high-salt-fed Wistar rats attenuated the levels of AT1R, PKCγ, and Rac1 activity and superoxides in the PVN, increased PVN antioxidant capacity, and decreased elevated BP [34]. The PVN microinjection of PKCγ siRNA had the same effect on hypertension as that of losartan, but had no effect on AT1R level in the PVN. These results indicate that Rac1 plays an important role in RAS-regulating ROS generation in the PVN and is involved in developing salt-sensitive hypertension via the AT1R/PKCγ/Rac1 pathway [34]. A high-salt diet elevated the Na^+^ of the CSF in DS rats but not in their salt-resistant or normotensive counterparts [85]. According to Blaustein et al., in salt-sensitive rats, the high Na^+^ of CSF-evoked locally secreted hypothalamic ouabain, aldosterone, Ang II, ENaC, and ouabain-sensitive α2 Na^+^ pumps also participate in a pathway that mediates sustained increase in SNA [82]. Ouabain increases AT1R and NADPH oxidase in the hypothalamus and decreases neuronal nitric oxide synthase (NOS) protein expression [82,90]. The aldosterone-ENaC-ouabain-α2 Na^+^ pump-Ang II pathway is involved in the regulation of BP set point and SNA via cardiovascular control centers in the brain [82].

## 5. Vascular Smooth Muscle Cell

### 5.1. Role of Rho in Vascular Smooth Muscle Contraction and the Mechanism of Rho-Associated Salt-Sensitive Hypertension

The vascular tone is under the control of various neural, humoral, and rheological stimuli, in response to which intracellular calcium concentration (Ca^2+^) increases as the key event in vascular smooth muscle excitation-contraction coupling [60]. VSMC contraction is ultimately caused by the phosphorylation of the myosin light chain (MLC) in contractile machinery (Figure 1). The phosphorylation of MLCs is induced by two mechanisms: the increment of cytosolic Ca^2+^ concentration (intracellular Ca^2+^ entry) and Ca^2+^ sensitivity. MLC is regulated by phosphorylation by Ca^2+^/calmodulin-dependent MLC kinase (MLCK), while dephosphorylation by Ca^2+^-independent MLC phosphatase (MLCP) and MLCP activity is inhibited by activated Rho/ROCK [60]. A number of vasoactive peptides, such as norepinephrine, acetylcholine, serotonin, Ang II, endothelin-1 (ET-1), and thromboxane A2 (TXA2), activate G proteins G_q_ and G_11_ via their respective G-protein-coupled receptors (GPCRs), which in turn activate phospholipase C to induce Ca influx into the cell, which results in Ca2+- and calmodulin-dependent activation of MLCK and subsequent phosphorylation of MLC, finally promoting vascular smooth muscle contraction [61]. Ang II, ET-1, and TXA2, through their respective GPCRs, also activate G proteins G_12_ and G_13_, and further activate the Rho/ROCK pathway, inhibiting MLCP and in turn promoting MLC phosphorylation, and ultimately contributing to vascular smooth muscle contraction by increasing Ca^2+^ sensitivity [62,63]. The activation of RhoA through G_12_ and G_13_ is mediated by a subgroup of Rho GEFs that are activated through direct interaction with Gα_12_ and Gα_13_ [64]. In the process of intracellular Ca^2+^ entry, GPCRs stimulated by vasoconstrictors activate phospholipase C (PLC), producing second messenger inositol triphosphatase (IP_3_) and diacylglycerol (DAG). PLC-induced IP_3_ production stimulates intracellular Ca^2+^ release from the sarcoplasmic reticulum, and DAG activates protein kinase C (PKC). PKC activates numerous plasma membrane Ca^2+^ channels and promotes Ca^2+^ influx and increased intercellular Ca^2+^ concentration [91,92]. Ca^2+^ binds to calmodulin, and its complex induces a conformational change in MLCK, converting it from an inactive to an active state in the contractile machinery [93]. On the other hand, increased Ca^2+^ sensitivity in VSMCs is caused by the inhibition of MLCP activity to sustain the phosphorylation of MLC.

The Ca^2+^ sensitization of myosin involves ROCKs (ROCK1, ROCK2), which are serine/threonine kinases and downstream effectors of RhoA. RhoA, abundantly expressed in VSMCs, is rapidly activated by vasoconstrictors such as Ang II and activates ROCK. ROCK stimulates the phosphorylation of myosin phosphatase target subunit 1 (MYPT1), a regulatory subunit of MLCP, at T696 or T853 [94]. MYPT1 phosphorylation, in turn, interferes with binding MLCP to MLC and accordingly sustains the phosphorylation of MLC, leading to contraction. In addition to MYPT1, ROCK phosphorylates C-kinase-potentiated protein phosphatase 1 inhibitor molecular mass 17 kDa (CPI-17), a smooth muscle-specific inhibitor of MLCP [65]. The DAG-PLC-PKC pathway also phosphorylates CPI-17. Thus, the activation of RhoA/ROCK is involved in the increment of Ca^2+^ sensitivity in VSMC and the vascular tone, which also affects the pathogenesis of hypertension and cardiovascular-renal disease [50]. Although several vasoconstrictors, such as Ang II, ET-1, and TXA2, have been shown to activate Rac1, the role of Rac1 in the regulation of VSMC tone is less understood than that of RhoA [95]. Rac1 inhibits MLCK phosphorylation via its effector p21-activated kinase 1 (PAK1), thus antagonizing the action of RhoA and negatively regulating VSMC tone [96].

There are three possible mechanisms of Rho-related salt-sensitive hypertension in vascular smooth muscle cells (Figure 1). These include (1) the stimulation of VSMCs by increased responsible vasoconstrictors such as Ang II, ET-1, and TXA2 in the blood, and (2) abnormal activations of Rho GEFs, and aberrant activation of Rho/ROCK by, for example, Wnt noncanonical pathway in aging. These, via Ca^2+^ influx into the cells (1) and increased Ca^2+^ sensitivity (1~3), induce VSMCs contraction and increase vasoconstriction, resulting in salt-sensitive hypertension. The detailed mechanisms are discussed below.

### 5.2. Rho GEF-Related Salt-Sensitive Hypertension

Experimental models of hypertension exhibiting vascular RhoA/ROCK activation are augmented with a high-salt diet [97]. A number of mechanisms implicated that RhoA/ROCK hyperactivation in hypertension includes the dysregulation of Rho GEFs [98]. On the other hand, the relationship between hypertension and Ang II is deep and aggravated by salt intake [15,99]. The chronic infusion of Ang II induces a gradual increase in BP in rats and mice [100]. Ang II-induced BP elevation is caused by the action in vascularity, central nervous system, and kidneys to modulate vascular resistance and neurohumoral pathways involved in sympathoexcitation, vasopressin release, and renal tubular water and salt absorption [76,96]. In these mechanisms, the activation of RhoA/ROCK plays a crucial role, especially in the vasculature [42,72,96].

Guilluy et al. showed that RhoA GEF Arhgef1 is specifically responsible for the Ang II-induced activation of RhoA signaling in arterial smooth muscle cells and essential for Ang II-induced hypertension [42]. They identified that Arhgef1 is specially activated by Ang II among Rho GEFs, and found that Ang II activates Arhgef1 through the activation of tyrosine kinase Jak2, inducing the phosphorylation of Tyr738 in Arhgef1. They generated a mouse model specifically lacking Arhgef1 in smooth muscle cells (SM-Arhgef1–/–) and demonstrated that SM-Arhgef1–/– mice showed resistance to Ang II-dependent hypertension but did not affect normal BP regulation. NOS inhibitory L-NAME-induced hypertension is also totally abolished in SM-Arhgef1–/– mice, but deoxycorticosterone acetate (DOCA) salt-induced hypertension is only partially suppressed. These results suggest that the activation of the AT1R-Arhgef1-RhoA pathway in smooth muscle cells is essential for Ang II- and L-NAME-induced hypertension but partially involved in DOCA salt-induced hypertension.

Wirth et al. showed that the activation of Arhgef12 (LARG)-RhoA is essential for DOCA salt-induced hypertension [72]. They generated a mouse model’s smooth muscle cells specifically lacking in G_q_-G_11_ (SM-q-11-KO) and G_12_-G_13_ (SM-12-13-KO). The mean BP of SM-q-11-KO significantly dropped, whereas those of wild-type (WT) and SM-12-13-KO remained at normal levels. However, BP was not elevated in either SM-q-11-KO or SM-12-13-KO by DOCA salt treatment, while DOCA salt induced remarkable BP elevation in the WT. These results indicated that the regulation of basal BP requires only G_q_-G_11_-mediated signaling in VSMCs, but both G_q_-G_11_- and G_12_-G_13_-mediated signaling are crucially involved in the elevation of BP in salt-sensitive hypertension. G proteins G_12_ and G_13_ activate the Rho/ROCK pathway via Rho GEF proteins p115-Rho GEF (Arhgef1), PDZ-Rho GEF (Arhgef11), and LARG (Arhgef12) [64], and LARG was the most predominant in the media of mouse aorta [72]. Then, when LARG-deficient mice were treated with DOCA salt, they did not develop hypertension, though they showed no abnormalities in basal BP. These data showed that the G_12_-G_13_-mediated activation of Rho/ROCK via the Rho GEF protein LARG is a central mechanism of vascular smooth muscle tone regulation in salt-sensitive hypertension. In addition, they showed that α1-adrenergic effector phenylephrine on smooth muscle cells is exclusively mediated by G_q_-G_11_, whereas the effects of other vasoconstrictors, such as Ang II, ET-1, or TXA2, are also mediated by G_12_-G_13_. Furthermore, the relatively strong antihypertensive effects of ET-1 and TXA2 antagonists in DOCA salt-hypertensive mice compared to normotensive mice suggested that ET-1 and TXA2 play an important role in the mechanism of salt-sensitive hypertension [72].

## 6. Kidney

### Crosstalk between RhoA and Rac1 in Salt-Sensitive Hypertension

**Figure 2:**
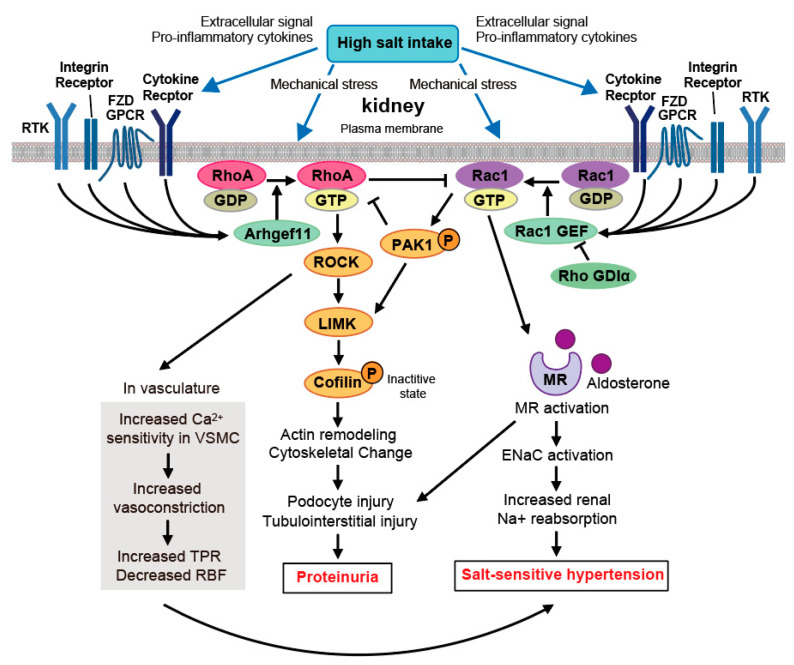
Cross talk between RhoA and Rac1 in salt-sensitive hypertension and renal injury in the kidney of Dahl salt-sensitive (DS) rats. In DS rats, a high-salt diet causes salt-sensitive hypertension and exacerbates renal damage, which is closely related to the activation of RhoA and Rac1 in the kidney. Various extracellular stimuli activate Rho GTPases via their receptors such as G-protein coupled receptors (GPCRs) [115], receptors of the tyrosine kinase (RTKs) [116], cytokine receptors [117], and integrin receptors [118] as well as Wnt and Notch signaling [119,120] by activating specific guanine nucleotide exchange factors (GEFs) [121]. Mechanical stress also stimulates RhoA and Rac1 [122]. It is known that salt intake increases the production of cytokines such as IL-1β and IL-17 in the blood, resulting in salt-sensitive hypertension [123] and that cytokines activate Rac1 [117]. In addition, Wnt5a is produced by macrophages and adipocytes via paracrine and autocrine upon external stimuli and further promotes cytokine production and secretion [124]. While Wnt5a is involved in enhancing inflammation, it activates RhoA [13,125]. These findings suggest that salt intake stimulates cytokine and Wnt protein production from macrophages and tissues and activates Rac1 and Rho, although the detailed mechanism of Rac1 and RhoA activation by high-salt diet is still unknown. In the kidneys of high-salt-fed DS rats, RhoA is activated by Arhgef11, which is activated by salt intake, and ROCK and LIM-kinase (LIMK) are sequentially activated, and cofilin activity is suppressed, resulting in actin polymerization and remodeling in glomerulus tubules and interstitium leading to marked proteinuria and tubulointerstitial injury. In the vasculature, activated ROCK increases vasoconstriction through increased Ca^2+^ sensitivity in VSMCs, increases total peripheral resistance (TPR), and decreases renal blood flow (RBF), thereby contributing to the development of salt-sensitive hypertension. On the other hand, salt intake activates Rac1 by Rac1-GEF, which synergistically activates mineralocorticoid receptors (MR) with aldosterone and increases sodium reabsorption via epithelial sodium channel (ENaC) in the renal collecting duct, resulting in salt-sensitive hypertension with glomerular and tubulointerstitial injury. Rac1 activates downstream p21-activated kinase 1 (PAK1), but PAK inhibits RhoA, and RhoA inhibits Rac1. Since PAK activates LIMK, there is LIMK-mediated crosstalk between RhoA and Rac1 in the DS kidney, and both are involved in the development of salt-induced renal injury.

In addition to LARG, Rho GEF Arhgef11 is involved in salt-sensitive hypertension [48]. The DS rat, a commonly used genetic model to study salt-sensitive hypertension [32,101,102], develops hypertension and kidney injury, including glomerulosclerosis, tubulointerstitial fibrosis, and vascular hypertrophy, and kidney injury becomes more severe as BP increases with a high-salt diet. In contrast, spontaneously hypertensive rats (SHRs) are resistant to hypertension-associated kidney injury compared to DS rats [103]. Garrett et al. conducted genetic studies using DS and SHR rats and identified at least nine loci throughout the genome linked to kidney injury [104]. Furthermore, Williams et al. narrowed the genomic region to a handful of genes and identified Arhgef11 as the top candidate for underlying the genomic locus through positional cloning using congenic strains and recombinant progeny testing [103]. They generated specific DS rats with SS-Arhgef11^SHR^, having the SHR allele substituted for the DS allele of Arhgef11 and showing reduced Arhgef11 expression, and demonstrated that SS-Arhgef11^SHR^ significantly decreased proteinuria, fibrosis, and improved renal hemodynamics without impacting BP compared to the control DS rats [103]. DS rats showed increased protein expression of Arhgef11, RhoA, and its downstream proteins ROCK1, LIM-kinase (LIMK) 1, and p-cofilin in the renal cortex, and significantly increased proteinuria despite having a low-salt diet compared to SS-Arhgef11^SHR^. Proteinuria in DS rats was suppressed to the same extent as that in SS-Arhgef11^SHR^ by Rho-kinase inhibitor fasudil, whereas that in SS-Arhgef11^SHR^ was not affected by fasudil. Under low salt, proteinuria in SS-Arhgef11^SHR^ was significantly lower than that in control DS rats, but similarly increased with weeks of age, differing from SHRs, which almost never present proteinuria [103]. These results suggest that Arhgef11-RhoA activation is important for the development of renal damage in DS, but other factors may be involved in developing kidney injury. Following these experiments, an Arhgef11 knockout DS rat (*Arhgef11* KO) was recently generated [48]. After 8 weeks of high- or low-salt diets, DS rats had significantly increased BP and intense proteinuria in the high-salt group compared to in the low-salt group, whereas *Arhgef11* KO had unchanged BP and increased proteinuria in the high-salt group, but it was controlled to about 35% of the DS high-salt group. RNA sequencing and discovery proteomics on the kidneys of DS and *Arhgef11* KO (on low salt) rats revealed the loss of Arhgef11 initiating transcriptome/protein changes in the cytoskeleton that impact a number of cellular functions, including actin cytoskeletal regulation, mitochondrial metabolism, and solute carrier transporters [48]. This series of studies indicated that Arhgef11 is involved in salt-sensitive hypertension and salt-induced renal damage in DS rats, but suggested that other responsible molecules must be involved in kidney injury (Figure 2).

Regarding this, we reported that the Rac1-mediated activation of MR in the kidneys plays a central role in salt-sensitive hypertension and salt-induced renal injury in DS rats [32]. In a previous report, we showed that Rac1 can activate MR in an aldosterone-independent manner and that Rac1 activation in mice results in prominent renal damage via MR activation [31]. In DS rats, high salt intake activates Rac1-specific GEF Tiam1 and Rac1 sequentially, which are involved in hypertension and the exacerbation of renal damage via MR activation, while DR rats did not show Tiam1 and Rac1 activation, hypertension, and renal injury by high salt intake (Figure 2) [32]. When it comes to Rac1-associated renal disease, a type of human congenital nephrotic syndrome and treatment-resistant nephrotic syndrome have been identified that were caused by mutations in Rho GDIα [105,106]. Nephrotic syndrome shows prominent proteinuria because of malfunction of the kidney glomerular filter, including podocytes. It was shown that mutations in *ARHGDIA* (R120X, G173V) from individuals with treatment-resistant nephrotic syndrome were accompanied by active Rac1, but not RhoA [105]. ARHGDIA protein is rich in human podocytes, and ARHGDIA mutation and subsequential Rac1 activation is thought to be involved in increased actin remodeling in podocytes, which are seen in the nephrotic state. In other animal models and studies, Rac1 was activated by salt intake [15,107,108]. Furthermore, human-gene-based analyses revealed that the *RAC1* gene was significantly associated with BP elevation to high-sodium intervention [33]. Rac1 activation via Rac-GEF, which is induced by salt intake, is, therefore, a key player in regulating salt sensitivity and causes salt-sensitive hypertension and renal injury.

Rac1 can inhibit RhoA via PAK1, and RhoA can inhibit Rac1 [109], so there is a Rac-Rho mutually inhibitory crosstalk from the perspective of cell morphology. Double-negative feedback loops resulting from mutual inhibition can lead to bistability [110,111], and it explains that there is both RhoA and Rac1 activation in the kidneys of salt-fed DS rats. RhoA and Rac1 have the common downstream effector LIMK, which phosphorylates cofilin to induce actin skeletal remodeling [112,113], and LIMK activation in DS rats was observed with both RhoA and Rac1 activation (Figure 2) [48,114]. These findings suggest that RhoA and Rac1 have crosstalk, and their activations are involved in the development of salt-sensitive hypertension in a complementary manner.

## 7. Aging Vasculature

### 7.1. Aging-Associated Hypertension via Noncanonical Wnt-RhoA/PCP Signaling

**Figure 3:**
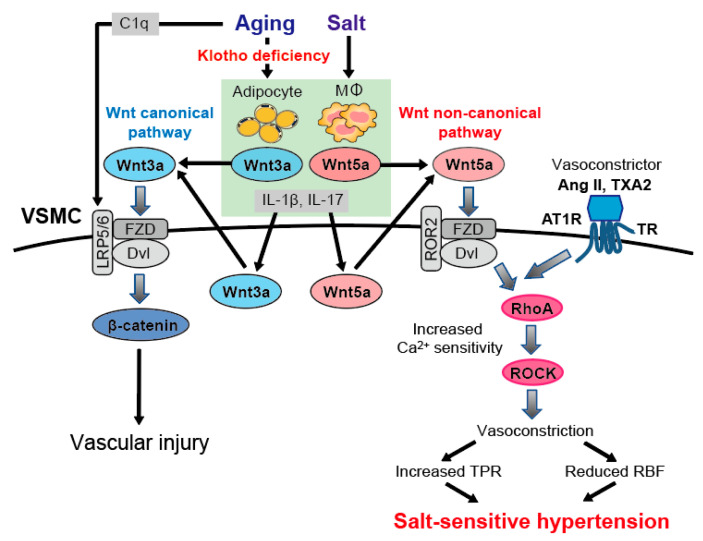
Potential mechanisms of aging-associated salt-sensitive hypertension and crosstalk between Wnt noncanonical pathway and angiotensin II (Ang II)-induced vasoconstrictive pathway. In aged mice with reduced serum klotho levels, high salt intake stimulates adipocytes and macrophages to produce cytokines such as IL-1β and IL-17, which in turn stimulate vascular smooth muscle cells (VSMCs) to secrete Wnt3a and Wnt5a. Inflammatory cytokines also upregulate Wnt5a expression in macrophages and adipocytes via paracrine and autocrine signaling. Secreted Wnt5a activates the noncanonical Wnt-RhoA (PCP) pathway by binding to its receptor Frizzled (FZD) and the Wnt coreceptor tyrosine-protein kinase transmembrane receptor ROR2 in VSMCs. Activated RhoA/Rho-associated protein kinase (ROCK) increases the Ca^2+^ sensitivity of VSMCs, leading to vasoconstriction. On the other hand, Ang II-induced RhoA/ROCK activation needs Wnt5a, and there is the crosstalk between Wnt noncanonical pathway induced- and Ang II-induced vasoconstriction in the process of RhoA activation. Consequently, total peripheral resistance (TPR) increases, and renal blood flow (RBF) decreases in the development of salt-sensitive hypertension. Aging-related salt-sensitive hypertension is thought to be related to increased vascular sensitivity to thromboxane A2 (TXA2) as well as Ang II. The canonical Wnt3a-β-catenin pathway is already enhanced with a normal salt diet, which might be a general feature of aging, regardless of klotho deficiency. Increased levels of complement C1q also activate the Wnt-β catenin pathway via cleavage of the Wnt coreceptors LRP5 and LRP6. High salt intake further increases Wnt3a-β-catenin signaling in the setting of klotho deficiency, leading to cardiac and vascular damage and arterial stiffness. Dvl, Dishevelled; LRP5/6, low-density lipoprotein receptor-related protein 5 and low-density lipoprotein receptor-related protein 6. We have modified this figure from our previous paper described below. This material is from: Wakako Kawarazaki and Toshiro Fujita, Kidney and epigenetic mechanisms of salt-sensitive hypertension, Nat Rev Nephrol, 2021, [Nature Springer] [37].

Wnt signaling is one of the fundamental mechanisms associated with many biological processes, including cell differentiation, proliferation, polarity, and migration; it regulates embryonic and adult stem-cell development and adult tissue homeostasis [126]. The Wnt signaling pathway plays a crucial role in vascular development and endothelial-cell specification [127] and is deeply involved in age-induced phenotypic changes such as atherosclerosis [128,129]. The activation of Wnt signaling causes senescence, and anti-aging factor klotho has potent anti-Wnt signaling activity [130]. Klotho is produced primarily in the kidneys but also in the parathyroid glands and the choroid plexus of the brain. It is secreted into the bloodstream, where circulating klotho acts on distant organs such as hormones [131]. Various epidemiological studies showed that the klotho protein in serum decreases with age [132,133]. An inverse relationship between circulating klotho and mean BP changes following salt loading was reported in patients with hypertension [134], suggesting that reduced levels of serum klotho contribute to salt-sensitive hypertension in the elderly. Klotho-deficient states such as aging promote the activation of Wnt noncanonical signaling and its downstream RhoA/ROCK and lead to salt-sensitive hypertension [13]. There are two major Wnt signaling pathways: the canonical β-catenin pathway and the noncanonical pathway, acting independently of β-catenin, and the former is better known (Figure 3). The Wnt ligand binds to transmembrane receptors of Frizzled (FZD). The activation of the Wnt canonical pathway stabilizes β-catenin, a transcriptional coactivator, and drives the expression of genes such as c-myc, cyclin D1, or PPARγ that support cell growth, proliferation, and survival [135]. The noncanonical pathway includes the Wnt/Ca^2+^ and planar-cell-polarity (PCP) pathways. The Wnt/Ca^2+^ pathway stimulates intracellular Ca^2+^ release, which, in turn, leads to the activation of PKC and Ca^2+^-calmodulin-dependent protein kinase II and affects cell adhesion and movement [136].

Signaling through the PCP pathway regulates cell polarity by controlling the actin cytoskeleton and asymmetric cytoskeleton formation [137]. In the PCP pathway, two independent pathways are initiated by Disheveled (Dvl), a downstream effector of FZD, and trigger the activation of Rho and Rac, respectively. In Rho-mediated signaling, Daam-1 forms a complex with Dvl and Rho and activates ROCK, whereas Rac-mediated signaling does not interact with Daam-1 and activates Jun kinase (JNK) [138]. However, how Dvl determines which of these two pathways to activate is only partly understood.

The human and mouse genomes contain 19 Wnt genes encoding highly conserved cysteine-rich secreted glycoproteins [128]. Among them, Wnt3a and Wnt5a are representative agonists that activate the canonical Wnt β-catenin and noncanonical Wnt-RhoA (PCP) pathways, respectively. On a normal salt diet, Wnt3a canonical signaling is upregulated in the vasculature of aged mice, unlike the WT. However, it was not enhanced in young klotho heterozygous knockout mice even though serum klotho was reduced to the same extent as in aged mice.

This indicates that the Wnt3a canonical pathway is upregulated with age, independent of serum klotho reduction. When fed a high-salt diet, aged mice and klotho heterozygous knockout mice, unlike WT mice, showed salt-sensitive hypertension, vascular RhoA activation, and increased vasoconstriction. At the same time, in both aged mice and klotho heterozygous knockout mice, the high-salt diet increased the expression of Wnt3a and Wnt5a in the vasculature, and the Wnt3a β-catenin and Wnt5a-RhoA pathways were both enhanced. To determine which pathway is involved in the development of salt-sensitive hypertension, the β-catenin inhibitor ICG-001 and the Wnt inhibitor LGK974, which suppresses both Wnt pathways, were administered to aged mice and klotho heterozygous knockout mice with a high-salt diet. Only Wnt inhibitor LGK974 suppressed vascular RhoA activation and vasoconstriction in these high-salt-fed mice, preventing the development of hypertension, and Rho inhibitor fasudil showed a similar effect to that of LGK974. These indicate that the noncanonical Wnt5a-RhoA pathway is involved in salt-induced BP elevation in the setting of klotho deficiency [13].

Furthermore, the gene transfer of the mouse klotho gene into aged and klotho heterozygous knockout mice using viral vectors restored serum klotho to the same level as that in young WT mice and suppressed vascular RhoA activation and salt-induced elevated BP.

These results indicated that high dietary salt intake under serum klotho deficiency contributes to salt-sensitive hypertension via activating the noncanonical Wnt5a-RhoA pathway in the vasculature and explained the mechanism of aging-associated hypertension (Figure 3) [13]. Though the mechanism of how cells receive the information of dietary salt intake is not fully elucidated, several lines of evidence indicate that inflammatory cytokines, including IL-1β and IL-17 that are upregulated in response to a high-salt diet, contribute to the development of salt-sensitive hypertension [123], and that IL-1β activates Rho/ROCK through Wnt5a expression upregulation [120]. Moreover, inflammatory cytokines and Wnt5a are abundantly expressed in macrophages and visceral adipose tissue [124,139]. The stimulation of salt intake might be received by inflammatory cells to produce IL-1β and IL-17, which stimulates macrophages and adipocytes to secrete more cytokines and Wnt5a, and induces the upregulation of Wnt5a in VSMCs (Figure 3).

### 7.2. RBF Reduction via Ang II-Wnt5a-RhoA Activation in Aging-Associated Hypertension

In the mechanism of salt-sensitive hypertension, decreased RBF is an important mechanism for decreasing renal sodium excretion. Decreased RBF is caused by increased renal vascular resistance, which is due to the hypercontractility of renal blood vessels and inadequate relaxation involving abnormal vascular endothelial function [9,140,141,142]. In response to a high-salt diet, RBF increases in normotensive and salt-resistant hypertensive individuals but not in salt-sensitive hypertensive individuals due to increased renal vascular resistance, which impairs renal sodium excretion and contributes to the development of hypertension [9,143,144]. In fact, in elderly hypertensive subjects, the increase in BP due to salt intake is associated with increased heterogeneous vascular resistance in various vascular beds, including the renal arteries [145]. In addition, since decreased NO production is associated with decreased RBF in aged rats and is ameliorated by RAAS inhibitors, it is suggested that aberrant activation of the RAS in aging kidneys is responsible for decreased RBF via decreased NO production [141,142]. In aged and klotho heterozygous knockout mice, high salt intake activated the noncanonical Wnt-RhoA pathway of renal arteries, contributing to the development of salt-sensitive hypertension by increasing sensitivity to Ang II and enhancing vasoconstriction [13]. Ang II, a potent vasoactive substance, causes the strong contraction of renal arteries and modulates RBF and BP [22]. In VSMCs, Ang II both promotes Ca^2+^ entry and increases Ca^2+^ sensitivity via the activation of RhoA/ROCK [42,62]. In an in vitro study, small-interfering RNA knockdown of Wnt5a in cultured VSMCs abolished Ang II-induced increases in RhoA activity, but additional Wnt5a canceled the effect of Wnt5a and brought Ang II-induced RhoA activation [13]. Moreover, klotho inhibited RhoA activation induced by Ang II or Wnt5a in these cells. These results indicated that Wnt5a is indispensable for Ang II-induced activation of RhoA/ROCK, and klotho worked to antagonize Wnt5a. There is the crosstalk between Wnt-noncanonical pathway induced- and Ang II-induced vasoconstriction in the process of RhoA/ROCK activation (Figure 3) [13]. In an in vivo study, the injections of Ang II or TXA2 analog U46619 into a renal artery induced vasoconstriction and remarkable reduction in RBF in aged and young klotho heterozygous knockout mice on high-salt diets, resulting in increased BP [13]. However, klotho supplementation ameliorated these effects and prevented the development of salt-induced hypertension in these mice. Thus, in aging that is accompanying decreased soluble klotho, salt intake causes the activation of the noncanonical Wnt5a-RhoA pathway, and reduced RBF via vasoconstriction of renal arteries contributes to the development of age-related salt-sensitive hypertension. As the mechanism of antagonizing Wnt5a by klotho, soluble klotho directly binds to the Wnt protein and facilitates a conformational change in the binding cavity of Wnt receptor FZD, inhibiting the Wnt protein from binding to FZD and thus Wnt signaling [146].

## 8. Endothelium

### Vascular Endothelial Dysfunction by Rho/ROCK Activation

Endothelial dysfunction is one of the important mechanisms in the development and progression of salt-sensitive hypertension [12,147,148]. Since NO is a potent vasodilator, decreased bioavailability of NO in the vasculature causes endothelial dysfunction, leading to increased endothelial permeability, platelet aggregation, leukocyte adhesion, and cytokine production [149]. Animal studies have shown that endogenous NO is critical for regulating renal hemodynamics and sodium homeostasis, including renal vasodilation and natriuresis [12]. Clinical data suggest that salt-sensitive patients are unable to upregulate NO production in response to salt intake [12]. NO in the vasculature is primarily produced by endothelial NOS (eNOS), the major isoform of NOS, which is a series of enzymes that oxidize L-arginine to L-citrulline and NO. Reductions in the expression or activity of eNOS, and its uncoupling, which enhances oxidative stress by decreasing NO and increasing O_2_ and peroxynitrite production, have a crucial role in the process of endothelial dysfunction [150].

In endothelial cells, the RhoA/ROCK pathway negatively regulates NO production and function through multiple mechanisms [96]. First, the activation of RhoA/ROCK decreases eNOS expression by reducing eNOS mRNA stability [151]. Second, RhoA/ROCK also suppresses eNOS function via the inhibition of the phosphoinositide 3-kinase (PI3K)/Akt/eNOS pathway [152]. Akt, a serine/threonine protein kinase, is the key downstream effector of PI3K. Akt can directly phosphorylate eNOS and activate it, leading to NO production. The active RhoA/ROCK pathway inhibits eNOS phosphorylation and cellular NO production via the suppression of Akt activation [153]. PI3K-dependent Akt activation can be regulated through tumor suppressor phosphatase and tensin homolog (PTEN) working in the opposite way as that of PI3K. RhoA/ROCK can also suppress the PI3K/Akt/eNOS pathway via the direct activation of PTEN [154]. Third, the activation of RhoA/ROCK by ROS suppresses NO production and reduces NO bioavailability. When superoxide reacts with NO, peroxynitrite is produced. Peroxynitrite oxidizes and reduces tetrahydrobiopterin (BH4), a cofactor required for eNOS activity and NO synthesis, ultimately resulting in decreased NO production. Peroxynitrite also increases active RhoA in models of experimental diabetes [155]. Fourth, arginase enhances RhoA/ROCK and diminishes NO level and eNOS bioavailability. Arginase, which is a hydrolytic enzyme that converts L-arginine into urea and ornithine, competes with NOS for their common substrate, L-arginine, leading to a decrease in NO. In a diabetic animal model, both ROS and arginase contributed to vascular endothelial dysfunction by decreasing eNOS bioavailability via RhoA/ROCK activation [155]. Fifth, Rho activation via symmetric dimethylarginine (ADMA), an endogenous inhibitor of NOS, decreases NO production. ADMA is associated with endothelial dysfunction and is an independent risk factor in the pathophysiology of salt-sensitive hypertension [12,14,45,156]. ADMA is endogenously synthesized during the methylation of protein arginine residues by protein arginine methyltransferases (PRMTs). There are two major types of PRMTs: PRMT-1 catalyzes the formation of ADMA, while PRMT-2 is involved in the formation of symmetrical dimethylarginine (SDMA), which has no eNOS inhibitory activity. Recent studies showed that ADMA activates RhoA and inhibits Rac1 in endothelial cells in vivo and in vitro [157]. ADMA influences Rho GTPase activity via NO and its downstream effector, PKG. A decrease in NO production by ADMA leads to the inhibition of PKG-mediated phosphorylation of RhoA at Ser^188^ in endothelial cells, resulting in activating RhoA and inhibiting Rac1 [157]. In vitro, a high-salt medium significantly increased ADMA concentration, PRMT-1 and RhoA expression, and activated ROCK, and eNOS expression had been suppressed [45]. In a study of African-Americans, the baseline of serum ADMA levels was similar in salt-sensitive and -resistant objects, but serum ADMA levels were significantly elevated in salt-sensitive objects compared to those in salt-resistant objects with a high salt intake and were involved in developing salt-sensitive hypertension through an increase in systemic vascular resistance [14]. These findings indicate that, in salt-sensitive individuals, salt intake causes an increase in serum ADMA levels, and a decrease in eNOS expression and NO production, though the increase in BP is also involved in no small part in this pathogenesis [158]. Thus, salt intake and increased salt sensitivity are associated with the development of hypertension, increasing ADMA production, decreasing NO production, and promoting RhoA activation. RhoA activation then induces RhoA-dependent stress fiber formation and enhances cell-substratum adhesion, which reduces endothelial-cell motility and angiogenesis, and causes various cardiovascular disorders such as hypertension, atherosclerosis, diabetes, and hypercholesterolemia [159].

## 9. Potential Role of Rho as a Therapeutic Target in Salt-Sensitive Hypertension

There are two isoforms of Rho kinase, ROCK1 and ROCK2, which are highly homologous. Rho kinases are involved in important physiological functions of cells such as regulation of smooth muscle cell contraction, migration, proliferation, maintenance of cell viability and morphology by regulating stress fibers and focal adhesions, and induction of gene expression. Therefore, Rho is deeply related to diseases such as hypertension, cerebro-cardiovascular diseases, cancer, and erectile dysfunction, and ROCK inhibitors are expected to be new therapeutic agents for these diseases. Fasudil, a Rho-kinase inhibitor, was the first protein phosphatase inhibitor to be approved and marketed in the world. Fasudil was launched in 1995 for the treatment of cerebral vasospasm. Fasudil is used for the treatment and prevention of cerebral vasospasm and cerebral ischemia, which is often caused by subarachnoid hemorrhage [160]. In addition, ROCK inhibitors are clinically indicated for the treatment of glaucoma [161,162] and have also been shown to be effective in the treatment of pulmonary hypertension [163,164]. Fasudil is a pan-ROCK inhibitor that inhibits both ROCK1 and ROCK2, and it is approved for use in Japan and China, but not by the United States Food and Drug Administration or the European Medicines Agency. Because of the pervasive and important role of ROCK in cytoskeletal dynamics and intracellular signaling, there have been concerns that the use of pan-ROCK inhibitors may cause cytotoxicity and other adverse effects. With this in mind, selective ROCK inhibitors and soft ROCK inhibitors have recently been developed. Since soft drugs are designed to maximize target organ exposure and minimize systemic exposure, they are already biologically active compounds that undergo rapid metabolic conversion to predictable non-toxic species as they enter the systemic circulation [165]. Soft ROCK inhibitors are a good indication, for example, for topical glaucoma eye drops [166].

ROCK, together with Akt and p70S6K, belongs to the AGC (protein family A, G, C) kinases, which are serine/threonine kinases. These kinases are involved in cancer cell motility, metastasis, survival, and immunity, and are considered to be important anticancer drug targets. AT13148, an oral AGC kinase inhibitor, is a potent inhibitor of ROCKI/II and Akt kinases and has been shown to inhibit cancer metastasis and growth in preclinical studies. In the first human clinical trial with AT13148 in 51 patients with solid tumors, dose-limiting toxicities included hypotension (300 mg), pneumonia, elevated liver enzymes (240 mg), and skin rash (180 mg), and the most common side effects were fatigue, nausea, headache, and hypotension [167]. In other words, dose-limiting toxicities were mainly due to ROCK inhibition (hypotension and headache) and not Akt inhibition (hyperglycemia and rash). Although 180 mg was the maximum tolerated dose of the drug, post-treatment biopsies of 3 of 8 patients showed a decrease of more than 50% in p-cofilin. In the end, AT13148 was not recommended for further development due to its narrow therapeutic potential area [167]. This study may serve as a reminder that systemic administration of broad-spectrum, potent AGC inhibitors can have various and strong adverse effects.

Psoriasis is caused by an abnormal IL-23/Th17-dependent immune response [168]. On the other hand, oral administration of KD025, a selective ROCK2 inhibitor, to healthy subjects was known to reduce the secretion of IL-17 and IL-21 [169]. In a clinical trial of a selective ROCK2 inhibitor in patients with psoriasis vulgaris, psoriasis area and severity index scores were reduced by 50% from baseline in 46% of patients, and epidermal thickness and T-cell infiltration of the skin were reduced [170]. A significant decrease in IL-17 and IL-23 was observed in patients, but there were no changes in other cytokines or significant adverse effects. Thus, the oral selective ROCK2 inhibitor improved the clinical symptoms of psoriasis patients by down-regulating the target Th17-driven autoimmune response without any adverse effects despite its systemic administration. The effects on BP and the cardiovascular system would have to be seen at higher doses. Thus, selective ROCK inhibition may be one of the important ways to reduce side effects, although the effects of ROCK inhibitors may vary depending on the indicated disease and target tissue as well as the selectivity of ROCK inhibition.

In a clinical trial by Masumoto et al. comparing forearm vascular resistance in hypertensive and normotensive patients, it was significantly higher in the former, but when fasudil was infused into the arteries of the patients, it decreased significantly more in the hypertensive patients [171]. In contrast, forearm vasodilation by sodium nitroprusside was comparable between the two groups. These results indicated that activation of ROCK was involved in the pathogenesis of increased peripheral vascular resistance in human hypertension. Results in animal models suggest that increased Ca^2+^ sensitivity of vascular smooth muscle mediated by ROCK occurs in salt-sensitive hypertensive patients and may be involved in the increase in systemic vascular resistance [15,72].

Taken together, ROCK inhibitors may be a promising therapeutic target for salt-sensitive hypertension. In the future, it is expected to develop therapies that are more selective for ROCK isoform and do not cause adverse effects other than BP suppression.

## 10. Conclusions

In the modern world, the amount of salt intake is high, which acts as a common cause of salt-sensitive hypertension. Dietary salt-induced BP elevation is the vital mechanism in evolution to maintain life against dehydration and desiccation and is organized by multiple organs and systems. Small GTPases Rho and Rac, which are activated by dietary salt intake, play important roles in the pathogenesis of salt-sensitive hypertension through various mechanisms as key switches of intracellular signaling. In the central nervous system, Rho is involved in RAAS activation and increases sodium sensitivity and sympathetic nerve outflow in BP control centers. In VSMCs, Rho GEF and Rho determine sensitivity to various vasoconstrictors, including potent vasoconstrictive substance Ang II. Meanwhile, they are involved in vasoconstriction via the G-protein and Wnt pathways regulating MLC phosphorylation in contractile machinery, leading to increased renal vascular resistance and decreased RBF. In the kidney, RhoA is involved in the formation of renal injury by altering the cytoskeleton through opposition and crosstalk with Rac1. In the vascular endothelium, Rho inhibits NO production and function, causing aberrant relaxation and increased vascular tone. All these mechanisms are involved in the development of salt-sensitive hypertension. For example, RhoA expression is increased in aging vessels, and klotho deficiency and high salt intake trigger noncanonical Wnt-Rho activation; klotho replacement therapy, Wnt inhibitors, and Rho-kinase inhibitors are expected to be new therapies in aging-associated hypertension [13]. The mechanism of how cells receive information on dietary salt intake and develop hypertension is not fully elucidated. However, if it can be clarified, it would help to develop new preventive and therapeutic strategies to control both salt-sensitive hypertension and cerebrocardiovascular and renal diseases in which salt is deeply involved. Chronic inflammation occurs in aged bodies, and the decreased anti-aging factor correlates increased salt sensitivity with aging. Moreover, salt intake induces the production of proinflammatory cytokines and enhances inflammation. Considering these things, salt intake is associated with various chronic diseases and aging. Understanding these propositions and grasping the role of Rho, regulating cell construction and motility would aid us in controlling salt sensitivity to prevent disease and aging in the future.

## Data Availability

Not applicable.

## Abbreviations

Ang II	Angiotensin II
BH4	tetrahydrobiopterin
BP	blood pressure
Cdc42	cell division control protein 42 homolog
CSF	cerebrospinal fluid
CPI-17	C-kinase potentiated protein Phosphatase 1 inhibitor, molecular mass 17 kDa
DAG	diacylglycerol
DOCA	deoxycorticosterone acetate
DS	Dahl salt-sensitive
ENaC	epithelial sodium channel
ET-1	Endothelin-1
eNOS	endothelial nitric oxide synthetase
FZD	Frizzled
GAP	GTPase-activating protein
GDI	GDP-dissociation inhibitor
GEF	Guanine nucleotide exchange factors
GDP	Guanosine diphosphate
GTP	Guanosine triphosphatase
GPCR	G-protein-coupled receptor
IP3	inositol triphosphatase
ICV	intracerebroventricular
JNK	c-Jun N-terminal kinase
LIMK	LIM-kinase
MR	mineralocorticoid receptor
MLC	myosin light chain
MLCK	myosin light chain kinase
MLCP	myosin light chain phosphatase
MYPT	myosin phosphatase target subunit
NO	nitric oxide
NOS	nitric oxide synthetase
NTS	nucleus of the solitary tract
PCP	planar-cell-polarity
PI3K	phosphoinositide 3-kinase
PLC	phospholipase C
PKC	protein kinase C
PRMT	protein arginine methyltransferases
PTEN	phosphatase and tensin homolog
PVN	paraventricular nucleus
RAAS	renin-angiotensin-aldosterone system
RAS	renin-angiotensin system
RBF	renal blood flow
RVLM	rostral ventrolateral medulla
ROCK	Rho-associated protein kinase
SDMA	symmetrical dimethylarginine
SFO	subfornical organ
SNA	sympathetic nerve activity
SNS	sympathetic nerve system
SON	supraoptic nucleus
TPR	total peripheral resistance
TXA2	thromboxane A2
VSMC	vascular smooth muscle cell
